# Regional differences in the pattern of airway remodeling following chronic allergen exposure in mice

**DOI:** 10.1186/1465-9921-7-120

**Published:** 2006-09-21

**Authors:** Jeremy A Hirota, Russ Ellis, Mark D Inman

**Affiliations:** 1 Firestone Institute for Respiratory Health, Department of Medicine, McMaster University, Hamilton, Ontario, Canada

## Abstract

**Background:**

Airway remodeling present in the large airways in asthma or asthma models has been associated with airway dysfunction in humans and mice. It is not clear if airways distal to the large conducting airways have similar degrees of airway remodeling following chronic allergen exposure in mice. Our objective was to test the hypothesis that airway remodeling is heterogeneous by optimizing a morphometric technique for distal airways and applying this to mice following chronic exposure to allergen or saline.

**Methods:**

In this study, BALB/c mice were chronically exposed to intranasal allergen or saline. Lung sections were stained for smooth muscle, collagen, and fibronectin content. Airway morphometric analysis of small (0–50000 μm^2^), medium (50000 μm^2^–175000 μm^2^) and large (>175000 μm^2^) airways was based on quantifying the area of positive stain in several defined sub-epithelial regions of interest. Optimization of this technique was based on calculating sample sizes required to detect differences between allergen and saline exposed animals.

**Results:**

Following chronic allergen exposure BALB/c mice demonstrate sustained airway hyperresponsiveness. BALB/c mice demonstrate an allergen-induced increase in smooth muscle content throughout all generations of airways, whereas changes in subepithelial collagen and fibronectin content are absent from distal airways.

**Conclusion:**

We demonstrate for the first time, a systematic objective analysis of allergen induced airway remodeling throughout the tracheobronchial tree in mice. Following chronic allergen exposure, at the time of sustained airway dysfunction, BALB/c mice demonstrate regional differences in the pattern of remodeling. Therefore results obtained from limited regions of lung should not be considered representative of the entire airway tree.

## Background

The hallmarks of asthma are variable airflow limitation associated with increased airway responsiveness, airway inflammation, and airway remodeling [1-5]. Ongoing airway inflammation and associated airway remodeling are believed to play a role in the development of airway hyperresponsiveness and airflow limitation. The relative contribution of various pathologic components to the increased airway responsiveness is yet to be elucidated, although airway remodeling appears to play a major role [3-5]. In human studies, advances in this area have relied on quantifying established airway remodeling and relating this to airway function measured at the same time [1,3,6]. In animal studies, greater insight is potentially afforded by observing the development of airway remodeling over time and relating this to changes in airway function occurring over the same period [7,8]. We currently use a murine chronic allergen exposure protocol that results in airway remodeling and associated sustained airway dysfunction which persists for up to 8 wks following cessation of allergen [7]. In human and animal approaches, assumptions have been made that measurement of airway remodeling changes at a single, or limited number of airway generations represents the whole lung. While this assumption is necessary when the access to multiple sites is limited (i.e. human biopsy studies), it is unlikely to be valid. In fact, there is evidence that the extent of specific indices of airway remodeling differs depending on the airway generation [9-11].

The involvement of the airways distal to the large conducting airways in respiratory disease, has been debated since Weibel's anatomical classification of small airways as being less than 2 mm in diameter [9,10,12-14]. More recently, the perception of the contribution of the small airways to overall lung resistance has shifted from a silent or quiet zone [15,16], to a more functionally relevant tissue [11,17].

To fully understand the contribution of each airway generation to airway disease we will require methods to assess inflammatory and structural changes throughout these airways. Similar to humans, the distribution of airway remodeling in mice following chronic allergen exposure is currently poorly described. We therefore felt it was prudent to develop and apply objective methods of quantifying airway remodeling throughout the tracheobronchial tree in animal models of allergic airway disease.

It is our hypothesis that quantifying the extent of several indices of airway remodeling in a range of airway calibers will reveal distinct patterns of changes at different levels of the tracheobronchial tree. To test this hypothesis, we present and characterize methods for assessing allergen-induced airway remodeling in the small and medium airways of mice having been subjected to chronic allergen exposure [7]. After optimizing these methods, we report that following chronic allergen exposure, distinct patterns of airway remodeling exist in different sized airways.

## Materials and methods

### Animals

Female BALB/c wild type mice, aged 10–12 weeks, were purchased from Harlan Sprague Dawley (Indianapolis, IN). All mice were housed in environmentally controlled, specific pathogen-free conditions for a one week acclimatization period and throughout the duration of the studies. All procedures were approved by the Animal Research Ethics Board at McMaster University, and conformed to the NIH guidelines for experimental use of animals.

### Sensitization and exposure

**Figure 1:**
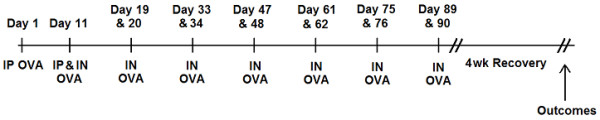
Chronic allergen exposure protocol. Sensitization was performed on Day 1 and Day 11. Six 2-day periods of allergen exposure, each separated by 12 days, started on Days 19 and 20. Outcomes were performed 4 wks post chronic allergen exposure.

Mice were sensitized as described previously by us [7]. Briefly, all mice received intraperitoneal (IP) injections of ovalbumin (OVA) conjugated to aluminium potassium sulfate on Days 1 and 11 and intranasal (IN) OVA on Day 11. Following sensitization, mice were subjected to a chronic allergen exposure protocol (Figure 1). Chronic allergen exposure was comprised of six 2-day periods of intranasal ovalbumin (IN OVA) administration (100 μg in 25 μl saline), each separated by 12 days. Exposures started on Days 19 and 20. Outcome measurements were made four weeks following the final period of allergen exposure and included (i) *in vivo *assessment of airway responsiveness to methacholine, (ii) large airway morphometry as described previously [18] (iii) a novel method for assessing morphometry of small and medium airways.

### Airway responsiveness

Airway responsiveness was measured by total respiratory system resistance (R_RS_) responses to intravenous saline and increasing doses of methacholine (MCh) using the FlexiVent ventilator system (n = 8 per group). Each mouse was anaesthetized with Avertin (2,2,2-Tribromoethanol, Sigma, Canada) via IP injection at a dose of 240 mg/kg and then underwent tracheostomy with a blunted 18-gauge needle, and then connected to the FlexiVent (SCIREQ, Montreal, Canada) computer-controlled small animal ventilator. Animals were ventilated quasisinusoidally (150 breaths/min, 10 ml/kg, inspiration/expiration ratio of 66.7%, and a pressure limit of 30 cmH_2_O). A script for the automated collection of data was then initiated, with the PEEP level set at 2 cmH_2_O and default ventilation for mice. After the mouse was stabilized on the ventilator, the internal jugular was cannulated using a 25-gauge needle. Paralysis was achieved using pancuronium (0.03 mg/kg intravenously) to prevent respiratory effort during measurement. To provide a constant volume history, data collection was preceded by a 6 sec inspiration to TLC perturbation (peak amplitude 25 cmH_2_O). Twenty seconds later the user was prompted to intravenously inject saline then 10, 33, 100, and 330 mg/kg of MCh (ACIC [Can], Brantford, ON, Canada). For each dose, thirteen "QuickSnap-150" perturbations (single inspiration/expiration of 0.4 sec duration with a volume amplitude relative to weight of 10 ml/kg) were performed over a 45 sec period, followed 10 sec later by another 6 sec TLC. After the last dose was complete, the mouse was removed from the ventilator and killed via terminal exsanguinations and subjected to further tissue collection. Airway responsiveness was quantified by the slope of the linear regression between peak respiratory system resistance and the log_10 _of the MCh dose, using the data from the 10, 33, and 100 μg/kg doses only. Heart rate and oxygen saturation were monitored via infrared pulse oxymetry (Biox 3700; Ohmeda, Boulder, CO) using a standard ear probe placed over the proximal portion of the mouse's hind limb.

### Lung histology

**Figure 2:**
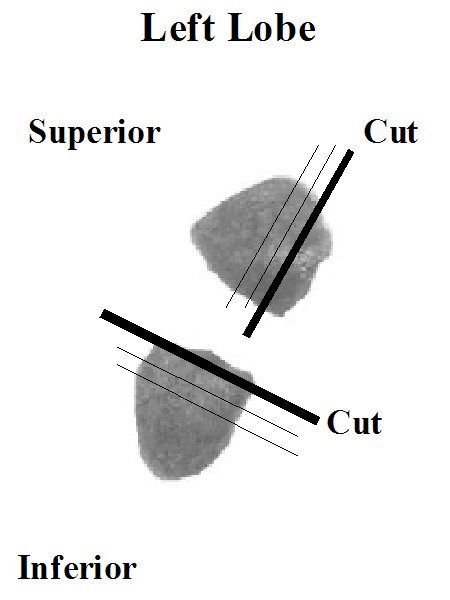
Depiction of left lobe following inflation and fixation with formalin. The left lobe was bisected to produce superior and inferior portions. The superior half of the left lobe was subjected to a sagittal cut. The superior and inferior portions were embedded in the same tissue block with extreme inferior and superior sagittal faces down (thick lines) and subjected to serially sectioning (fine lines).

Following *in vivo *assessment of airway responsiveness, lungs were dissected, removed, inflated with 10% formalin with a pressure of 25 cm H_2_O, ligated at the trachea, and fixed in 10% formalin for 24 hours. Following fixation, the left lung was isolated and bisected into superior and inferior segments (Figure 2). The inferior portion of the left lobe was embedded with the bisected face down to obtain transverse cross sections of the primary bronchus for large airway morphometry. The superior portion of the left lobe was subjected to a sagittal cut and embedded with the sagittal face down for airway morphometry of airways distal to the primary bronchus (Figure 2). Both superior and inferior lung portions were embedded in the same paraffin wax tissue block, and rough cut to expose a smooth tissue surface. Three micron thick sections were stained with Picrosirius Red (PSR) for assessing the presence of collagen. Further sections were immunostained using monoclonal antibodies against α-smooth muscle actin (α-SMA)(Clone 1A4, Dako, Denmark) and fibronectin (Clone 10, BD Biosciences, Canada)

### Lung morphometry

**Figure 3:**
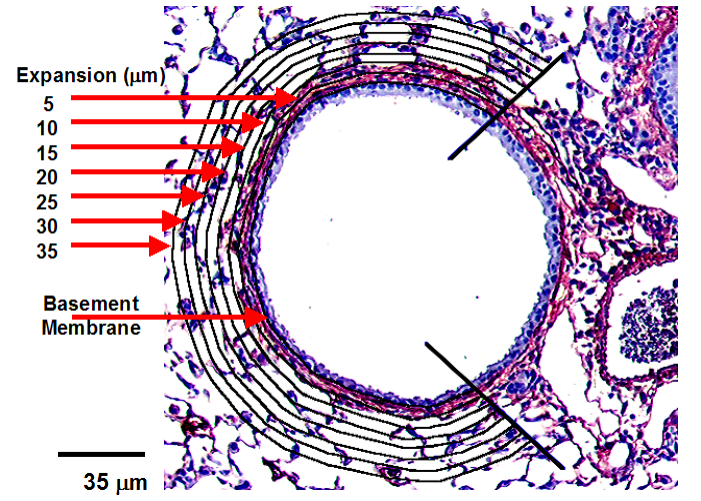
Depiction of a small airway captured for analysis. The airway is associated with vessels, which are excluded from morphometric analysis of airway walls. The sub-epithelial basement membrane of the airway wall free from vessel association is traced. A region of interest of defined band depth (5, 10, 15, 20, 25, 30, and 35 μm) is projected into the parenchyma from the sub-epithelial basement membrane trace (black lines). The stain of interest (α-SMA) is quantified by the software as a percentage of the total band area for each band depth.

All tissue sections were viewed and images collected under 20× objective magnification light microscopy (Olympus BX40; Carsen Group Inc., Markham Ontario). A customized digital image analysis system (Northern Eclipse, Version 7.0; Empix Imaging Inc., Mississauga, Ontario, Canada) with an attached digital pen and drawing tablet was used to collect and analyze images. Airways that satisfied the following criteria were included for airway analysis: (i) the airway needed to be completely contained in a single microscope field of view (690 μm × 520 μm); (ii) the ratio of the major and minor airway axes needed to be less than 2 (maximum diameter/minimum diameter) to ensure that the airway was not obliquely cut; (iii) the airway perimeter needed to be completely intact. Images of airways that satisfied these criteria were saved as tagged image file format files. Image collection and analysis was performed by two separate individuals; the first individual would collect, code, and determine the size of airways, the second individual would be blinded and analyze the collected coded images as follows. Using the custom digital image analysis system, quantification of the area of positive stain per region of interest was performed for α-SMA, PSR, and fibronectin stained tissues. Areas of airway wall associated with connective tissue from neighbouring vessels were excluded by drawing boundaries for analysis (Figure 3). While viewing the airway of interest, the basal border of the epithelium (corresponding to the basement membrane) was traced. The image with clearly defined boundaries for morphometric analysis was then saved as a new file to be used for all subsequent steps. Using the image file with established basement membrane trace, a 5 um thick region of interest extending from the trace out into the parenchyma was drawn using the digital pen and tablet (Figure 3). The software then calculated the area of stain within the region of interest based on previously determined stain specific colour plane settings. The amount of positive stain area was then expressed as a percentage of the region of interest area. The process was repeated for each airway image captured from the same animal, which were approximately 4 per animal. The average percent stain for all airways from the same animal was calculated and used for statistical analysis. The analysis on the same airway was repeated for 10, 15, 20, 25, 30, and 35 μm band depths. Medium and small airways were arbitrarily defined by determining the mean airway area of all airways collected. The airways with areas below the mean were defined as small, while airways with areas above the mean were defined as medium. Large airways were collected and analyzed as defined previously [18].

### Statistical analysis

Summary data used in all comparisons are expressed as mean and standard error of the mean (SEM). To determine optimum band depth for detecting airway remodeling changes, we calculated the sample size that would be required to demonstrate observed allergen-induced changes over a range of band depths. This was chosen as a practically useful way of identifying the band depth with optimal signal to noise characteristics. Sample sizes required for comparing two groups were estimated based on the difference of the means between the allergen and control groups and the mean value of the standard deviations at each given band depth. Sample size requirements were based on a Student's t test analysis and calculated with an assumed power of 80% (β = 0.2) and an α of 0.05. Differences were assumed to be statistically different when the observed p values were less than 0.05.

## Results

### Airway responsiveness

**Figure 4:**
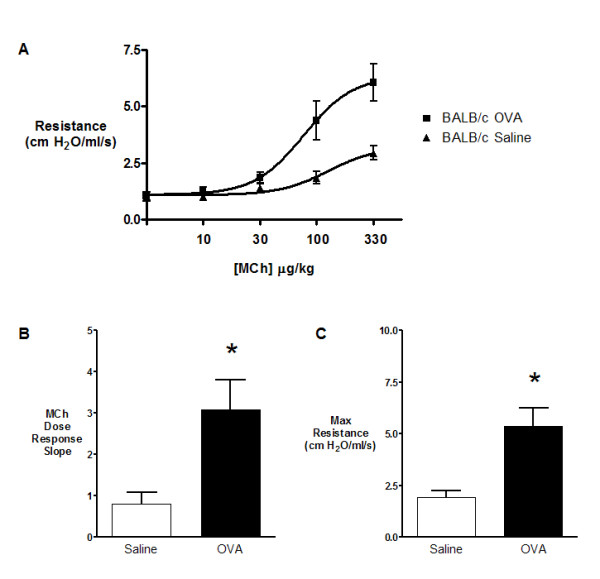
**A) **Airway physiology responses to increasing doses of MCh measured four weeks following chronic exposure to saline (open) or OVA (closed) on FlexiVent ventilator system. BALB/c saline (triangles), BALB/c OVA (squares). **(B) **Airway reactivity and **(C) **maximum respiratory resistance values as measured by MCh dose response slope and maximum resistance, respectively for chronic saline (open) or OVA (closed) BALB/c mice. Data are expressed as mean (SEM); 8 mice per group. * significantly different from corresponding control animals (p < 0.05).

Airway function measurements were made two weeks following chronic allergen exposure (Figure 4A). At this time point, significant increases in both airway reactivity and maximum R_RS _were observed in BALB/c mice as compared to control animals (p < 0.05; Figure 4B–C). Break point [7] and EC_50 _analysis of methacholine dose response curves revealed no changes in airway sensitivity (data not shown).

### Airway characteristics

Large (primary bronchus) airways used for airway remodeling analysis ranged from 212 760 μm^2 ^to 418 325 μm^2 ^in area. The mean airway area and diameter were 311 035 μm^2 ^and 630 μm, respectively. The airway ratio (maximum to minimum diameter) ranged from 1.02 to 1.95.

Airways distal to the primary bronchus used for airway remodeling analysis ranged from 12 269 μm^2 ^to 172 094 μm^2 ^in area. The mean airway area and diameter were 56 543 μm^2 ^and 270 μm, respectively. The airway ratio (maximum to minimum diameter) ranged from 1.01 to 1.98. The airways distal to the first generation bronchus were further divided into small (0–50 000 μm^2^) and medium (50 000 μm^2^–175 000) airways, based on mean area, for assessment of regional airway remodeling. The mean small and medium airway areas for saline and allergen exposed animals were not significantly different.

### Airway remodeling can be detected in airways distal from the primary bronchus of BALB/c mice

**Figure 5:**
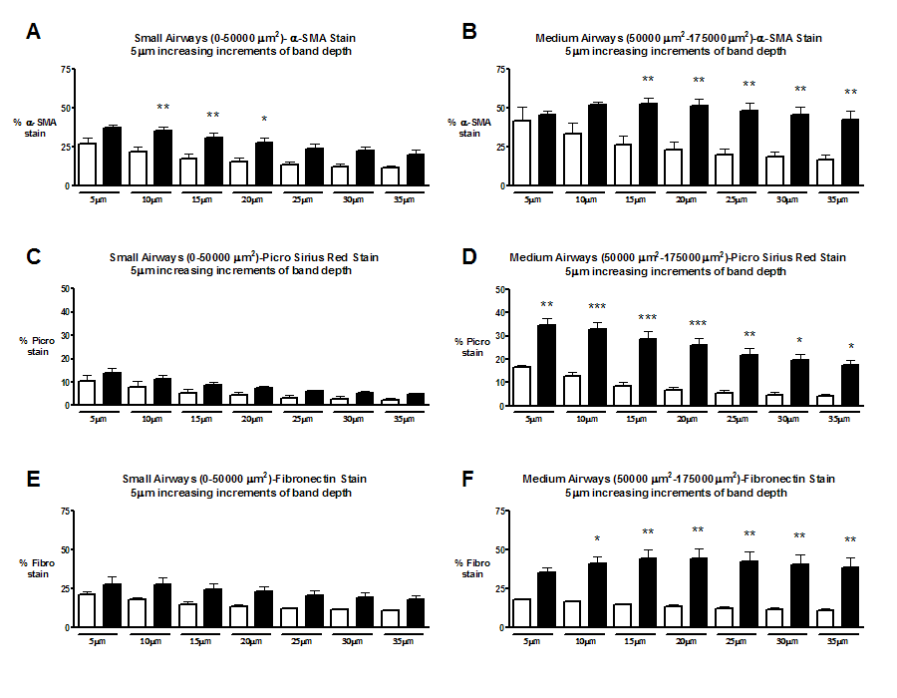
Morphometric analysis of small and medium airways following chronic exposure to saline (open) or OVA (closed). Morphometric analysis was performed at 5, 10, 15, 20, 25, 30, and 35 μm band depths. The stain of interest is expressed as a percentage of total band area. Open bars – saline exposed animals, Closed bars – ovalbumin exposed animals. **A) **Small airway α-SMA staining. **B) **Medium airway α-SMA staining. **C) **Small airway Picrosirius Red (PSR) staining. **D) **Medium airway PSR staining. **E) **Small airway fibronectin staining. **F) **Medium airway fibronectin stainingData are expressed as mean (SEM); 8 mice per group. * significantly different from corresponding control animals (p < 0.05). ** significantly different from corresponding control animals (p < 0.01). *** significantly different from corresponding control animals (p < 0.001).

Chronic intranasal allergen exposure resulted in a statistically significant increase in α-SMA content in the small and medium airways of BALB/c mice as compared to saline controls (Figure 5A–B). In small airways, the optimal band depth to detect α-SMA changes was 15 μm. This conclusion was based on the band width requiring the smallest sample size to detect the allergen-induced change in α-SMA content (Table 1). In medium airways, an allergen induced increase in α-SMA content was detected for band depths ranging from 15–35 μm (Figure 5B). In medium airways, the optimal band depth to detect α-SMA content changes was 20 μm (Table 1).

**Table 1:** Mean differences of percentage stain between saline and allergen exposed animals.

	**Band Depth (μm)**						
**Stain**	**5**	**10**	**15**	**20**	**25**	**30**	**35**
**α-SMA**							
Small	9.78 (9)	13.09 (5)	13.73 (4)	12.24 (5)	10.56 (6)	9.67 (6)	8.58 (6)
Medium	4.48 (84)	18.35 (6)	26.61 (4)	28.43 (4)	28.41 (4)	26.92 (4)	25.81 (4)
							
**PSR**							
Small	3.49 (44)	3.52 (34)	3.36 (20)	2.93 (18)	2.65 (14)	2.49 (12)	2.20 (11)
Medium	18.81 (3)	19.97 (3)	20.16 (3)	19.04 (3)	16.38 (3)	14.76 (3)	13.09 (3)
							
**Fibro**							
Small	6.66 (26)	9.67 (10)	9.59 (8)	9.42 (8)	8.76 (7)	8.16 (7)	7.40 (7)
Medium	16.92 (5)	24.66 (3)	29.55 (3)	30.76 (3)	29.88 (4)	28.79 (4)	27.20 (4)

Numbers in each column are absolute differences between mean values of percentage stain for saline and allergen exposed animals with sample size requirements for determining allergen induced effects in parenthesis.

α-SMA – α-smooth muscle actin stain

PSR – Picrosirius red stain

Fibro – Fibronectin stain

Allergen exposure did not result in statistically significant increases in PSR staining in the small airways (Figure 5C). The medium airways demonstrate statistically significant increases in PSR staining at all band depths assessed following chronic allergen exposure (Figure 5D). The optimal band depth to detect PSR changes was 15 μm (Table 1).

Allergen exposure did not result in statistically significant increases in fibronectin staining in the small airways (Figure 5E). Statistically significant increases in medium airway fibronectin content were detected following chronic allergen exposure (Figure 5F). The optimal band depth to detect fibronectin changes was 20 μm (Table 1).

### Regional differences in the pattern of airway remodeling are observed in BALB/c mice following chronic intranasal allergen

The data presented above illustrates differences in airway remodeling between small and medium airways. To further investigate the heterogeneity of airway remodeling we compared remodeling events between large (primary bronchus), medium, and small airways using optimized band depths (see above and ref [18]).

**Figure 6:**
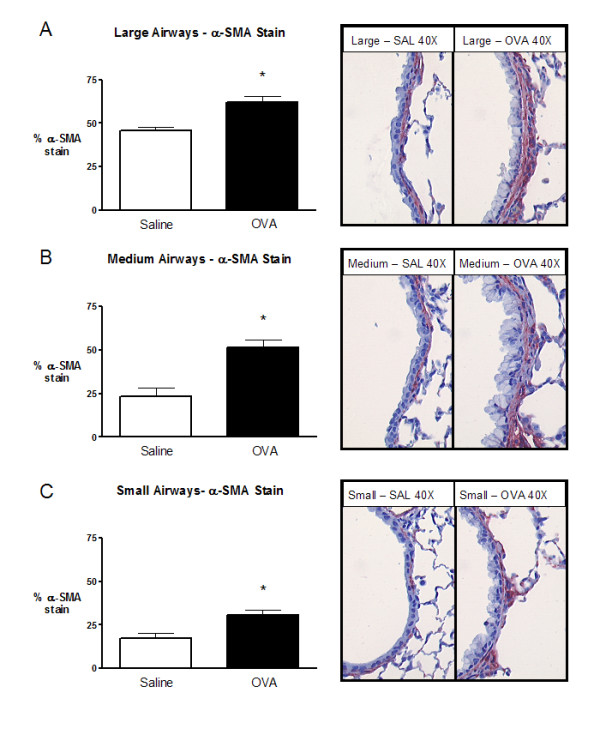
Morphometric analysis of smooth muscle content in small, medium, and large airways following chronic exposure to saline (open) or ovalbumin (closed). Morphometric analysis for small and medium airways used 15 and 20 μm band depths, respectively. Proximal airways were analyzed as described previously [18]. **A) **Large airway α-SMA staining. **B) **Medium airway α-SMA staining. **C) **Small airway α-SMA staining. Representative histology images for large, medium, and small airways are located to the right of the figures. Data are expressed as mean (SEM); 8 mice per group. * significantly different from corresponding control animals (p < 0.05).

Following chronic allergen exposure the medium airways demonstrated a 2.23 fold increase in smooth muscle content, compared to a 1.76 and 1.37 fold increase in the small and large airways, respectively (Figure 6).

**Figure 7:**
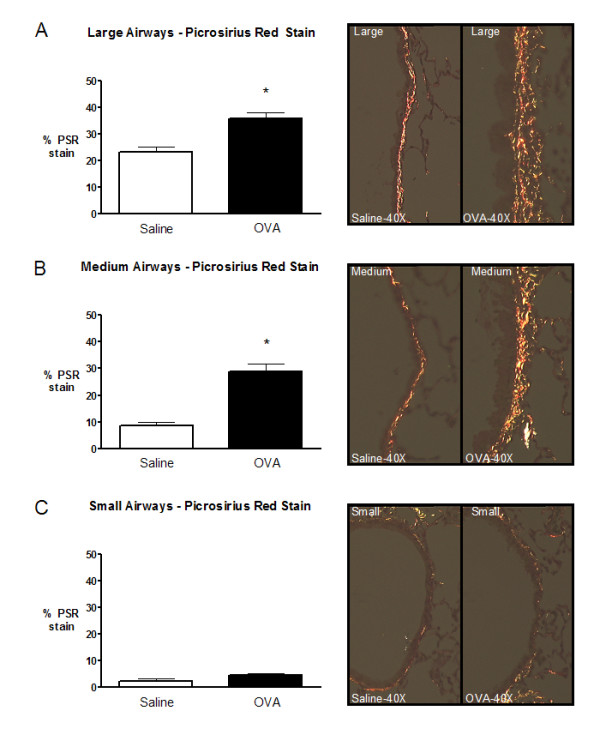
Morphometric analysis of collagen content in small, medium, and large airways following chronic exposure to saline (open) or ovalbumin (closed). Morphometric analysis for small and medium airways used 10 and 15 μm band depths, respectively. Proximal airways were analyzed as described previously [18]. **A) **Large airway PSR staining. **B) **Medium airway PSR staining. **C) **Small airway PSR staining. Representative histology images for large, medium, and small airways are located to the right of the figures. Data are expressed as mean (SEM); 8 mice per group. * significantly different from corresponding control animals (p < 0.05).

Similarly, there was a 3.31 fold increase in medium airway collagen content, compared to 1.87 and 1.72 fold increase in the small and large airways, respectively (Figure 7).

**Figure 8:**
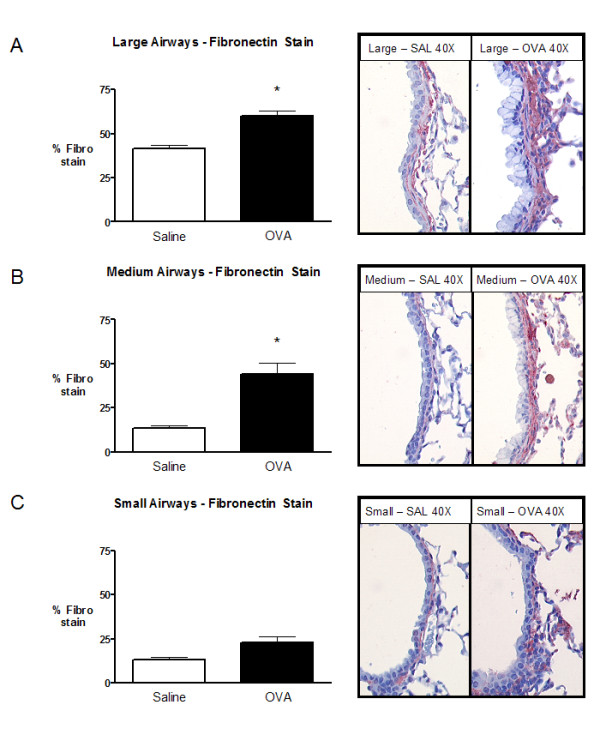
Morphometric analysis of fibronectin content in small, medium, and large airways following chronic exposure to saline (open) or ovalbumin (closed). Morphometric analysis for small and medium airways used 10 and 20 μm band depths, respectively. Proximal airways were analyzed as described previously [18]. **A) **Large airway fibronectin staining. **B) **Medium airway fibronectin staining. **C) **Small airway fibronectin staining. Representative histology images for large, medium, and small airways are located to the right of the figures. Data are expressed as mean (SEM); 8 mice per group. * significantly different from corresponding control animals (p < 0.05).

A 3.25 fold increase in fibronectin staining was observed in the medium airways, compared to 1.71 and 1.44 fold increase in the small and large airways, respectively (Figure 8).

## Discussion

Here we demonstrate that regional differences in the pattern of airway remodeling occur in the tracheobronchial tree of mice following chronic allergen exposure. Our morphometric methods for quantifying airway remodeling in mice is the first systematic airway remodeling analysis of the tracheobronchial tree following chronic allergen exposure. These findings are important in demonstrating that insults such as allergen can produce differential effects at different airway levels, which need to be considered when evaluating these animals. Our data therefore support the hypothesis that airway remodeling is heterogeneous in this model of allergen exposure. This emphasizes the importance of treating the tracheobronchial tree as being heterogeneous and argues against approaches with limited scope (e.g. biopsies) as being reflective of all airway generations.

It is important to emphasize that our decision to divide airways distal to the primary bronchus into small and medium airways is arbitrary and that no anatomical distinction should be inferred. Our division of airways into small, medium, and large groups is required to address the question of heterogeneous airway remodeling. Our findings should therefore be interpreted with this in mind. Precisely defining the airway size/environment required for specific remodeling events or the mechanism underlying these phenomenon was beyond the scope of this manuscript.

As we have previously established morphometric methods for evaluating allergen induced effects only in the large airways[18], we felt it was necessary to extend these techniques to smaller airways. In addition to demonstrating that significant allergen induced airway remodeling occurs in smaller airways, we show that intranasal allergen exposure results in distinct patterns of remodeling throughout the entire airway tree. The medium airways demonstrate the greatest fold increase in remodeling indices, as compared to the small and large airways. However, whether or not this is the site of the greatest functional consequences of airway remodeling is not known. Clearly, studies aimed at determining the individual contribution of small, medium, and large airways, as well as the specific remodeling events in these airways, to airway dysfunction are required.

We have observed distinct patterns of airway remodeling in different airway generations. While we have clearly demonstrated no statistically significant collagen remodeling in the small airways, it is likely that allergen induced changes in fibronectin would have been statistically significant with a greater sample size (as indicated in the Table). This suggests that studies should be powered according to each of the specific remodeling indices of interest. Failure to do this may result in Type II statistical errors and inappropriate interpretation of results.

Animal research ethics boards require strict guidelines for justifying the number of animals to be used in a given study. Funding agencies are increasingly interested in ensuring that studies are appropriately powered to detect the primary outcome of interest *a priori*. Our results demonstrate that distinct structural changes occur at different generations of airways, suggesting that group analysis of all airway sizes may mask a signal present in a particular airway size. To appropriately power studies, investigators should consider the sample size required for analysis of the specific airway size of interest.

The methods presented herein use a customized digital image analysis system, that consists of a CCD camera connected to a microscope and a computer. In addition to the hardware, software capable of detecting user defined colour plane settings is required. We feel that using our validation steps and producing an optimized morphometric technique could be of importance in other research areas including kidney fibrosis, gastrointestinal tract inflammation, and/or vascular biology.

In conclusion we demonstrate that distinct patterns of airway remodeling occur in the tracheobronchial tree of mice following chronic allergen exposure. These results demonstrate that the pathology observed in one area of the lung may not be representative of other regions. Clearly, future studies aimed at exploring structure-function relationships need to consider the heterogeneity of airway remodeling throughout the lung.

## Funding

Canadian Institutes for Health Research
